# Integrated Detection and Prediction of Influenza Activity for Real-Time Surveillance: Algorithm Design

**DOI:** 10.2196/jmir.7101

**Published:** 2017-06-15

**Authors:** Armin Spreco, Olle Eriksson, Örjan Dahlström, Benjamin John Cowling, Toomas Timpka

**Affiliations:** ^1^ Faculty of Health Sciences Department of Medical and Health Sciences Linköping University Linköping Sweden; ^2^ Department of Computer and Information Science Linköping University Linköping Sweden; ^3^ Department of Behavioural Sciences and Learning Linköping University Linköping Sweden; ^4^ WHO Collaborating Centre for Infectious Disease Epidemiology and Control School of Public Health The University of Hong Kong Hong Kong China (Hong Kong); ^5^ Region Östergötland Center for Health Services Development Linköping Sweden

**Keywords:** human influenza, algorithms, epidemiological surveillance, public health surveillance, evaluation research, epidemiological methods

## Abstract

**Background:**

Influenza is a viral respiratory disease capable of causing epidemics that represent a threat to communities worldwide. The rapidly growing availability of electronic “big data” from diagnostic and prediagnostic sources in health care and public health settings permits advance of a new generation of methods for local detection and prediction of winter influenza seasons and influenza pandemics.

**Objective:**

The aim of this study was to present a method for integrated detection and prediction of influenza virus activity in local settings using electronically available surveillance data and to evaluate its performance by retrospective application on authentic data from a Swedish county.

**Methods:**

An integrated detection and prediction method was formally defined based on a design rationale for influenza detection and prediction methods adapted for local surveillance. The novel method was retrospectively applied on data from the winter influenza season 2008-09 in a Swedish county (population 445,000). Outcome data represented individuals who met a clinical case definition for influenza (based on International Classification of Diseases version 10 [ICD-10] codes) from an electronic health data repository. Information from calls to a telenursing service in the county was used as syndromic data source.

**Results:**

The novel integrated detection and prediction method is based on nonmechanistic statistical models and is designed for integration in local health information systems. The method is divided into separate modules for detection and prediction of local influenza virus activity. The function of the detection module is to alert for an upcoming period of increased load of influenza cases on local health care (using influenza-diagnosis data), whereas the function of the prediction module is to predict the timing of the activity peak (using syndromic data) and its intensity (using influenza-diagnosis data). For detection modeling, exponential regression was used based on the assumption that the beginning of a winter influenza season has an exponential growth of infected individuals. For prediction modeling, linear regression was applied on 7-day periods at the time in order to find the peak timing, whereas a derivate of a normal distribution density function was used to find the peak intensity. We found that the integrated detection and prediction method detected the 2008-09 winter influenza season on its starting day (optimal timeliness 0 days), whereas the predicted peak was estimated to occur 7 days ahead of the factual peak and the predicted peak intensity was estimated to be 26% lower than the factual intensity (6.3 compared with 8.5 influenza-diagnosis cases/100,000).

**Conclusions:**

Our detection and prediction method is one of the first integrated methods specifically designed for local application on influenza data electronically available for surveillance. The performance of the method in a retrospective study indicates that further prospective evaluations of the methods are justified.

## Introduction

In light of the rapidly growing availability of “big data” from both diagnostic and prediagnostic (syndromic) sources in health care and public health settings, a new generation of epidemiological and statistical methods is needed for reliable analyses and modeling [1]. This need of new methods adapted to extensive but heterogeneous datasets extends to algorithms for detection and prediction of winter influenza seasons and influenza pandemics. Each year epidemics of influenza occur in communities worldwide and cause extensive morbidity and mortality [2,3]. Preparing for and responding appropriately to winter influenza seasons and pandemics is a critical function of public health. However, a concern regarding forecasting algorithms is that reports of methods used for analyses of extensive datasets originating from different sources do not always meet basic standards. The reports fail with regard to the requirement that evaluators should be able to assess the design and performance of the methods when building the next generation of algorithms [4]. However, regardless of the transparency problems in reporting, the potential of big data analyses in infectious disease control is widely recognized. For instance, autoregressive models for influenza forecasts have shown satisfactory performances when applied on large populations [5]. This implies that the area where the knowledge need presently is most immediate is the detection and prediction of influenza activity at local levels [6]. Such granular views, in turn, can provide input into large-scale models and accurate prediction of influenza spread in wide geographical areas.

Several weaknesses of infectious disease surveillance and prediction systems described in previous decades [7] have still not been addressed in methods design. Responding to this situation, the Centers for Disease Control and Prevention (CDC) in the United States initiated the “predict the influenza season challenge” [8], which encouraged researchers to forecast features of winter influenza season progression that are useful to policy makers and to take advantage of big data resources. For this competition, existing methods for modeling influenza were grouped into three categories:

(1) Compartmental models: These are based on mechanistic assumptions about how the influenza virus is transmitted and use these assumptions to estimate the number of individuals in various states related to a disease [9]. For instance, the Susceptible-Infectious-Recovered (SIR) model approximates the dynamics between groups susceptible to influenza, infected with the virus, and recovered from infection over time [10]. Assumptions typical for this category of models include that any pair of individuals in a defined group are equally likely to interact socially and that genetic subgroups within influenza strains behave identically.

(2) Agent-based models: These are more complex types of mechanistic models that typically use synthetic populations based on census data and build complex schemes of social interaction and disease progress in simulated individuals and communities [11,12]. These models can incorporate more detailed assumptions about transmission dynamics but can be computationally intensive.

(3) Nonmechanistic statistical models: These are phenomenological approaches, that is, they aim to model patterns and trends in the data without necessarily considering the underlying mechanisms. Typical approaches of this type are linear autoregression, which estimate influenza activity using a linear function based on recorded past activity. More complex methods in this category include generalized linear models, Box-Jenkins analysis [13], seasonal autoregressive integrated moving-average models [14], and generalized autoregressive moving- average models [15].

More alternative surveillance methods include, for instance, prediction markets [16] that combine expert predictions using a stock market-like system, and the method of analogues (k nearest neighbors) [17] that makes predictions of future influenza activity levels using similar patterns from the past without assuming a strict model. However, it is disconcerting that none of the above categories of forecasting methods evaluated in the CDC challenge generated satisfying results for all four aspects of influenza epidemics (ie, start week, peak week, peak percentage, and duration) [18].

The aim of this study was to present a novel method for integrated detection and prediction of influenza activity using data electronically available for real-time surveillance in local settings in the Western hemisphere and to evaluate its performance by retrospective application on authentic data from a Swedish county. By local settings in the Western hemisphere is meant communities with specified populations in Europe and North America. Winter influenza seasons and pandemics can be expected to spread to these settings, but the dissemination of the actual types and strains of influenza virus does not likely originate from there. In the presentation of the integrated detection and prediction method, the term epidemic is used as a summary label for both winter influenza seasons and pandemics.

## Methods

### Study Design

The novel method was formally defined based on a design rationale for integrated detection and prediction methods specifically adapted for application in local influenza surveillance. An overview of the method design is exhibited in the Results section, followed by detailed descriptions of the detection and prediction modules. The results of a retrospective performance study evaluation based on authentic data from a Swedish county are also presented. The study design was approved by the regional research ethics board in Linköping (dnr. 2012/104-31).

### Design Rationale

The rationale for design of a novel integrated detection and prediction method is that the aim of local influenza surveillance is early detection and prediction of infected individuals requiring clinical attention, with the purpose of timely allocation of scarce health care resources. Precious time is lost before laboratory data are available for algorithmic processing and test samples are not taken from all patients. Syndromic data are used for peak timing prediction because it is challenging to only use unidimensional gold standard data to predict the peak timing.

Both the detection and prediction functions are to comply with requisite quality and accuracy criteria for technologies to be used in health care and public health practice [19]. A theoretical assumption underpinning the design of the detection module is that the number of influenza cases grows exponentially in the beginning of periods with increased activity. Another assumption is that an alerting threshold can be determined using historical data from previous winter influenza seasons. For prediction of the peak timing, evidence of a strong association between the gold standard and syndromic data sources used for the surveillance is assumed to be available. For prediction of the peak intensity, the peak timing must have been determined and the influenza-diagnosis case rates must follow a bell-shaped function of time around the peak.

### Definitions

Influenza detection is defined as indicating the initiation of an epidemic in the community, that is, a prolonged period of elevated incidence rates (exceeding a given limit) of influenza cases, as defined by the rate of individuals clinically diagnosed with influenza in a population under surveillance. Influenza prediction denotes foretelling the peak timing and the peak intensity of an epidemic in the community. For detection, weekday effects and optimal alerting thresholds with reference to influenza-diagnosis data are retrospectively established in the method calibration. For prediction, both the weekday effects and the grouping of variables in the syndromic data with the largest correlation strength and longest lead time to influenza-diagnosis data are established.

The influenza case-rate level when a local influenza epidemic factually takes off was set to 6.3 influenza-diagnosis cases/100,000 during a floating 7-day period. This limit was determined by inspecting the epidemic curves of previous local influenza epidemics in the learning dataset. A similar definition (6.4 influenza-diagnosis cases/week/100,000) was determined for the winter influenza season in 2008-09 in a recent comparison of influenza intensity levels in Europe [20]. The definition of when an epidemic ends was set to the interepidemic (period between two epidemics) influenza-diagnosis level for the specific setting where the method is applied. This was done because the detection algorithm requires that the influenza activity is at an interepidemic level before the algorithm can start its search.

### Retrospective Performance Study

For a retrospective performance evaluation of the integrated detection and prediction method, outcome cases were represented by individuals clinically diagnosed with influenza during the 2008-09 winter influenza season in a Swedish county (population 445,000). The thresholds used in epidemic detection were determined using data from a learning dataset containing the 2008-09 winter influenza season. The metrics used to evaluate the detection of influenza epidemics were timeliness, sensitivity, and specificity. Timeliness was defined as the time difference (in days) between the actual start of the epidemic and the start indicated by the model. Specificity was calculated from when the detection algorithm is started (ie, when previous epidemic has come to an end) and until the beginning of the current epidemic per the standard definition (6.3 influenza-diagnosis cases/100,000 during a floating 7-day period). This means that the period length for specificity calculations varies with the interepidemic period. Sensitivity was calculated from the beginning of the current epidemic (according to the same definition) and 45 days into the epidemic. The optimal alerting threshold was decided by calculating sensitivity and specificity and studying them on a receiver operating characteristic (ROC) curve, giving specificity priority over sensitivity because a high level of false alarms is undesirable in public health practice.

To evaluate the prediction of the peak timing, timeliness (defined as time between the predicted day of the influenza-diagnosis peak (highest number of daily cases) and the day of the peak in the observed smoothed series (using moving average of influenza-diagnosis data) was used as metric. To evaluate the prediction of the peak intensity, the absolute and relative differences between the predicted peak intensity expressed as the number of influenza-diagnosis cases at the predicted day of the peak and the observed peak intensity were used as metrics. The reason for not comparing the predicted peak intensity with the actual peak intensity (ie, without smoothing data first) was to reduce the impact from possible outliers.

### Data Sources

Influenza cases were identified using the International Classification of Diseases version 10 (ICD-10) codes for influenza (J10.0, J10.1, J10.8, J11.0, J11.1, J11.8) [21] from the local electronic health data repository. For individuals having received an influenza-diagnosis at both primary and secondary levels of care, the diagnosis code recorded at the first contact was used for the analyses. If the codes were recorded at the same day, only the secondary-level diagnosis code was used. Correspondingly, information collected from the calls to a telenursing service in the county was used as syndromic data. Influenza-related telenursing call cases were identified by the chief complaint codes associated with influenza symptoms (dyspnea, fever [child and adult], cough [child and adult], sore throat, lethargy, syncope, dizziness, and headache [child and adult]) from the fixed-field terminology register. In accordance with Swedish legislation (SFS 2008:355), personal identifiers were removed from the records. In this study, only the chief complaints of fever in a child and adult were used because a previous study showed that this combination of complaints was most strongly associated with influenza diagnoses [22].

## Results

### Method Design Overview

**Figure 1:**
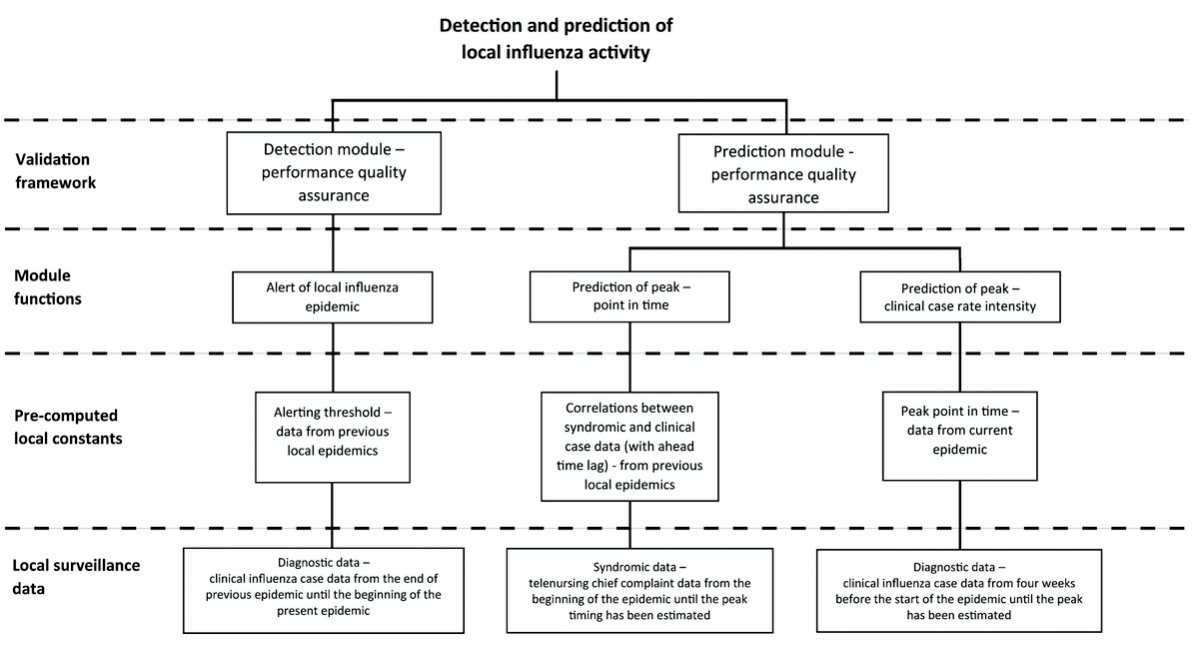
Structure of the integrated detection and prediction method displayed design patterns.

The integrated detection and prediction method is based on nonmechanistic statistical models, that is, patterns and trends in the data are modeled without necessarily considering underlying mechanisms. It is designed for integration in local health information systems. Accordingly, the underpinning structure is defined at four levels, ranging from data sources to performance validation (Figure 1). The method is divided into separate modules for detection and prediction of influenza activity, respectively. The function of the detection module is to alert for an upcoming period of increased load of influenza-diagnosis cases on local health care services, whereas the function of the prediction module is to predict the timing of the activity peak and its intensity. Early detection of increased influenza activity and prediction of peak intensity are based on streams of the gold standard data, whereas prediction of peak timing is based on syndromic data. In this setting, patients clinically diagnosed with influenza were used as gold standard.

**Figure 2:**
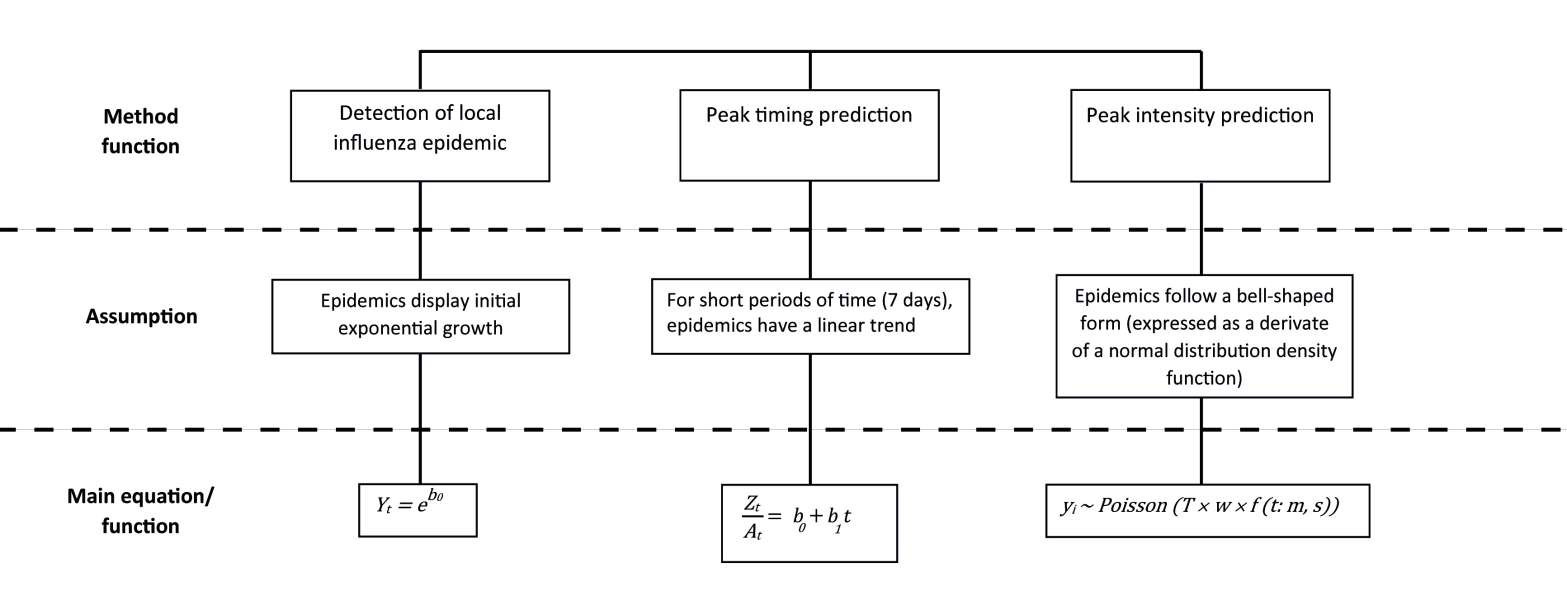
An overview of the main mathematical equations or functions used for each component.

An overview of the main statistical assumptions and equations for each component is displayed in Figure 2.

### Detection Module

Exponential regression (1) is used for detection modeling, based on the observation that the beginning of an influenza epidemic is assumed to have an exponential growth of infected individuals:

(1) *X*_t_*= e*^a₀ + b₁t^

with *t* representing the time, *a*_0_ representing the level, and *b*_1_ representing the trend. The expected number of visits at local health care services, *E[Y*_t_*]* is the product of *X* and the probability *p* for an infected individual to visit the local health care service. This expectation is also exponential in time:

(2) *E[Y*_t_*] = e*^a₀ + b₁t^*p=e*^a₀ + ln(p) + b₁t^*= e*^b₀ + b₁t^

Where *b*_0_ now combines the current level of number of infected and probability of visiting the local health care service without any possibility to separate them. As daily data are used in the analysis, weekday effects, *A*_w_, are also calculated and used as an offset variable in the exponential regression analysis. The weekday effects are calculated as follows: let *A*_Monday_ be the average number of events on Mondays during previous epidemics and denote the values for other weekdays by *A*_Tuesday_*, A*_Wednesday,_ and so on. Let *A*_Total_*=(A*_Monday_*+...+A*_Sunday_*)/7*. The multiplicative weekday effect for Mondays is *A*_Monday_*/A*_Total_ and so on. The weekday effects are included in the model:

(3) *E[Y*_t_*] = e*^b₀ + b₁t + ln(Aw)^

If *X* is large, *p* is small, and the infected individuals act independently, then *Y* is approximately Poisson distributed:

(4) *Y*_t_*~ Poisson (e*^b₀ + b₁t + ln(Aw)^*)*

Furthermore, the time is shifted, that is, the most recent day is considered as *t*=0, the second most recent day is considered as *t*=−1, and so on. For every new day, the time axis is moved one step so that the new “most recent day” is considered as *t*=0. For each day an exponential regression analysis (1) is run and a fitted value *ŷ* is calculated by inserting *t*=0 in equation (3) giving

(5) *Y*_t_*= e*^b₀^

as an estimate of the current level of visits which is smoothed for random variation and adjusted for weekday effects. This is repeated for each day by moving the time axis one day at a time so that the most recent point in time of the series is considered *t*=0. Doing this, one value is obtained for every day representing the level for that day. Finally, the lower 95% confidence limit is calculated to represent the level of influenza activity, which is then compared with a predetermined threshold. If the level is above the threshold, an alarm is raised, which means that the epidemic has started; and if the level is below the threshold, no alarm is raised.

Detection starts when the previous epidemic has ended (the interepidemic period level for the community where the detection component is applied), and runs during the inter-epidemic period until an increase in diagnosed influenza cases is detected. When the increase is confirmed, the algorithm is paused and restarted when the epidemic has ended.

The detection algorithm is adjusted in exceptional situations, that is, if an epidemic “simmers” before it begins. The risk of simmering is extensive for a pandemic or an exceptionally mild winter influenza season. In the first case, if there is a fear of a pandemic outbreak among the population, individuals are more likely to contact medical services for influenza symptoms, leading to an increased baseline which increases the risk for false alarms. Also, if a winter influenza season is exceptionally mild, individuals contacting medical services for influenza-like symptoms in the winter will sporadically be misdiagnosed with influenza before the actual circulation of the influenza virus, leading to an increased baseline and thus, an increased risk for false alarms. The alerting threshold determined in the learning set is therefore doubled in these particular cases. It was contended that a strong indication of preepidemic simmering is when it takes extended time between when the influenza incidence increases above a baseline level and when the start of the epidemic occurs (according to the standard definition 6.3 influenza-diagnosis cases/100,000 during a floating 7-day period). The definition for when the influenza incidence has increased above the baseline level is set to 3.2 influenza-diagnosis cases/100,000 during a floating 7-day period (ie, half of the start-of-epidemic definition). An epidemic is then defined to simmer if the time-period separating these 2 dates is longer than three times the average length of the period during previous local influenza epidemics. In other words, the alerting threshold is only doubled due to simmering if the incidence has increased over the baseline level but not exceeded the start-of-epidemic level during this observation period.

### Prediction Module

The prediction process is divided into two components. In the first component, syndromic data are used to predict the peak timing, and in the second component, influenza-diagnosis data are used to estimate the peak intensity.

#### Peak Timing Prediction

In the first component, the aim is to predict the peak timing using linear regression. Including weekday effects *A*_w_ and smoothed for random variation, the model for the number of cases in syndromic data is expressed as

(6) *Z*_t_*= (b*_0_*+ b*_1_*t) × A*_w_*,*

with *b*_0_ representing the level and *b*_1_ representing the trend. Since the weekday effects *A*_w_ are known, a model smoothed for weekday effects and random variation can be expressed as:

(7) *Z*_t_*/ A*_w_*= b*_0_*+ b*_1_*t*

For each 7-day period, a linear regression (7) is run and parameter estimates *b*_0_ and *b*_1_ are fitted. The idea is to estimate the trend in syndromic data for every 7-day period (the first period being days 1-7 and the second being days 2-8), from the beginning of an epidemic and until the peak is found. Although it is unlikely that an epidemic curve increases and decreases linearly, the assumption can be made that the trend during a short period of 7 days has almost a linear increase or decrease.

The search for the peak starts when the detection algorithm signals that an epidemic has taken off and continues until the peak is detected. To identify the peak timing, two conditions are set. As per the first condition, it is essential to ensure that the epidemic has a sufficiently sharp upward trend. The trend is therefore defined as sufficiently sharp when significantly positive (*P*<.05) trends *b*_1_ have occurred either during two consecutive or during three different 7-day periods. When one of these events has occurred, the second condition is applied. According to this condition, when the first significantly negative trend (*b*_1_) during a 7-day period has occurred, it is assumed that the peak has been reached on the first day of this period. However, there is a possibility that this 7-day period “overlaps” with a previous 7-day period, which includes a significantly positive trend. In that case, the first 7-day period with a significantly negative trend is ignored and the peak is instead assumed to appear during the second 7-day period with a significantly negative trend. The search is aborted if the peak is not found when the epidemic has already descended in the local setting where the algorithm is applied.

When the peak is found in the syndromic data, the 14 days preceding influenza-diagnosis data [22] is utilized to find the peak (in influenza-diagnosis data). In other words, if the peak in the syndromic data appears on day 0, the influenza-diagnosis peak is assumed to appear on day 14. However, it is possible that the peak in the syndromic data occurs on a day during the weekend but highly unlikely that the peak in influenza-diagnosis data occurs on one of these days as, for instance, primary care centers are closed during weekends in Sweden. Instead, it is reasonable to assume that the influenza-diagnosis peak occurs at the beginning of the week because individuals who suffer influenza symptoms during the weekend visit primary care centers when they reopen on Monday or possibly Tuesday. Adjustments are therefore made by moving the influenza-diagnosis peak to the following Monday if it is expected to occur on a Friday, Saturday, or Sunday according to syndromic data and to the previous Tuesday if the peak is expected to take place on a Wednesday or Thursday. If the peak is expected to occur on a Monday or Tuesday, no adjustments are made. In other words, in the first case the syndromic data precedes influenza-diagnosis data between 15 and 17 days, in the second case between 12 and 13 days, and in the third case 14 days.

Depending on what day of the week the peak in the syndromic data is expected to take place, the prediction of the influenza-diagnosis peak is made between 6 and 11 days before it is expected to occur, as the syndromic peak can be determined first after 6 days has passed of the syndromic data series.

#### Peak Intensity Prediction

In the second component of the prediction module, the aim is to predict only the peak intensity. Based on empirical assessments of previous epidemics, an epidemic adjusted for weekday effects is assumed to show a bell-shaped form from the beginning to the end, and can therefore be expressed using a derivate of a normal distribution density function. The intensity function must also include weekday effects and total number of events during the whole epidemic. Use of bell-shaped functions was systematically introduced in epidemiology by Brownlee in the early 20th century [23], and such functions have since then been applied in several contexts, for example, to predict the course of acquired immune deficiency syndrome (AIDS) in the United States [24]. Assuming that the peak timing is known (estimated in the first prediction component) and that an epidemic follows the bell-shaped function around the peak, the intensity function can be used to predict the peak intensity at time *m*.

Assume that day number *t*=1, 2, 3,..., *t*_i_; the observed number of influenza-diagnosis cases is *y*= *y*_1_, *y*_2_, *y*_3_,..., *y*_i_, and that

(8) *y~ Poisson (T × w × f (t: m, s))*

where *T* is the total number of health care visits of the whole epidemic, *w* is the weekday effects, *f* is the normal distribution density function, *t* is the day number, *m* is the center of the epidemic (which coincides with *t* for the peak), and *s* is the spread in time. Since *t*, *w*, and *m* are known, only the parameters *T* and *s* are estimated using *y* in such way so that the likelihood is maximized. However, in order to do that, first appropriate starting values for these parameters need to be selected. Finally, using the known parameter *m* and the estimated parameters *T* and *s*, the peak intensity at time *m* is calculated by replacing *t* with *m* in equation (8).

It is important that the start of the series seems appropriate because the second prediction component assumes that the level is zero or at an interepidemic level at the start and it is not optimal that there are single or occasional spikes at the beginning of the series. For that reason, the start of the series should be a couple of weeks before an epidemic is detected.

### Evaluation of Detection Module

**Figure 3:**
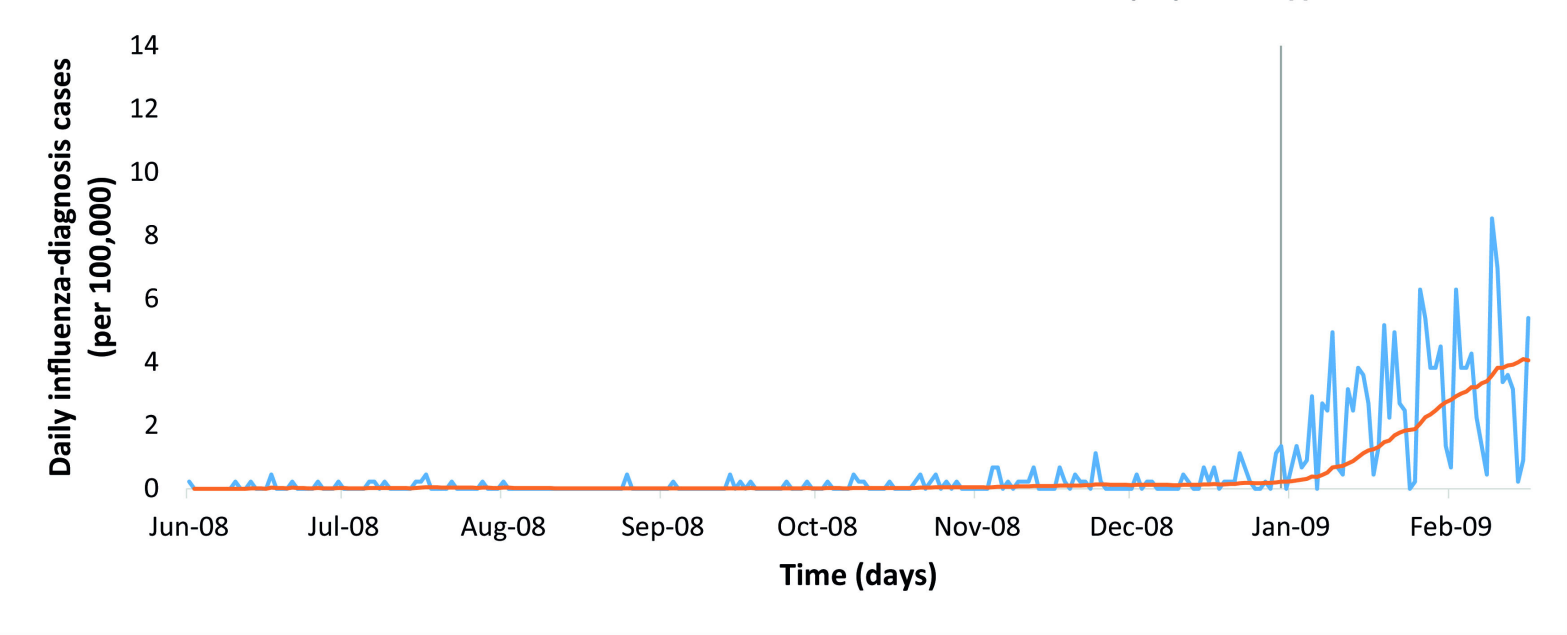
The detection algorithm applied on winter influenza season 2008-09 (A[H3N2]). The blue line represents the number of influenza-diagnosis cases/day/100,000, the gray bar marks the start of the winter influenza season according to the definition (6.3 influenza-diagnosis cases/100,000 during a floating 7-day period), and the orange line denotes the lower limit estimated using the detection algorithm.

The optimal threshold for the lower confidence limit of the expected number of influenza-diagnosis cases was computed to 0.21/day/100,000 for the detection algorithm. The detection sensitivity and specificity (calculation based on the interepidemic period 211 days) were both 1.00 and the timeliness 0 (Figure 3). This means that the detection module according to the definition (6.3 influenza-diagnosis cases/100,000 during a floating 7-day period) detected the 2008-09 winter influenza season on the day it actually started.

### Evaluation of Prediction Module

**Figure 4:**
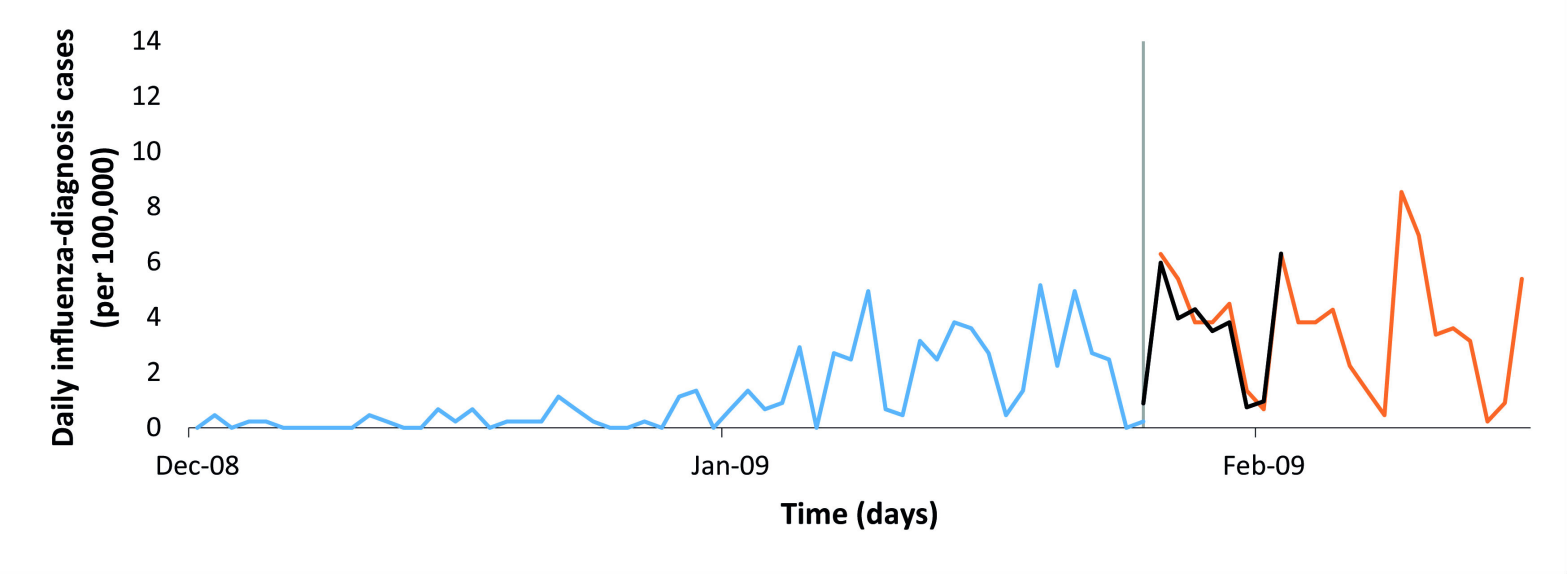
The prediction method applied on winter influenza season 2008-09 (A[H3N2]). The blue line represents the number of known actual influenza-diagnosis cases/day/100,000 at the time when the prediction is performed, the orange line represents the number of “unknown” actual influenza-diagnosis cases/day/100,000 at the time when the prediction is performed from the first unknown day and until the peak has passed, the gray bar marks the end of the known and the beginning of the “unknown” actual influenza-diagnosis cases/day/100,000, and the black line denotes the predicted values (using the peak intensity prediction) from the first “unknown” day and until the predicted peak occurs.

The prediction module performance was satisfying both with regard to the peak timing and peak intensity. The peak timing was estimated 8 days in advance and occurred 7 days before the factual peak occurred. The predicted peak intensity at the predicted day of the peak was estimated to 6.3 influenza-diagnosis cases/100,000 (Figure 4) compared with the factual 8.5 influenza-diagnosis cases/100,000 at the day of the actual peak, that is, the absolute difference between the predicted and the actual incidence was 26%.

## Discussion

### Principal Findings

The aim of this study was to present an integrated influenza detection and prediction method that uses data electronically available in local public health information systems for real-time surveillance. In the performance evaluation based on retrospective data, the method detected the winter influenza season of 2008-09 on the day it actually occurred, whereas the prediction module showed satisfying performance both with regard to the peak activity timing and its intensity.

### Comparison With Prior Work

Many important policy decisions in the response to increased influenza activity are made at the local level, for example, planning of resources at intensive care units and deciding social distancing measures such as school closures. The design of the presented integrated detection and prediction method can be compared with current state-of-the-art big data approaches to influenza forecasting [18]. One such approach for local application is a multi-linear, auto-regressive framework in which information is synthesized from a variety of data sources, ranging from Google Trends to electronic patient records (EPRs) [5,6,25-27]. In this study, clinical influenza-diagnosis cases were used for detection of a period of increased influenza activity and for prediction of the peak intensity, whereas syndromic telenursing data were used for prediction of the peak timing. We chose not to use data directly from EPRs due to integrity and legal issues. In addition to telenursing data, several other syndromic data sources were available that also displayed satisfactory associations with clinical influenza-diagnosis data, for example, Google Trends data, website use data, and local media coverage data [22]. Among the data sources available, telenursing and Google Trends data had the longest lead times and showed the strongest correlations with influenza-diagnosis data. However, since daily Google Trends data could not be guaranteed to be available on a constant routine basis, only telenursing data were used as syndromic data source in the present study. Moreover, we employ full-season learning periods for model updates, whereas continuous updating of the model during an epidemic is used in the multi-linear auto-regressive framework. We chose seasonal updates because a central design prerequisite was to highlight transparency of the method construction and thus facilitate implementation by other researchers. Adjustments of the algorithm processes are only made before pandemics and during winter influenza seasons to adjust detection levels for simmering low influenza activity. We gather that methods for continuous updating have to be dependent on the characteristics of local data and it is challenging to formally define a continuous updating framework in a decontextualized format sufficiently transparent to allow transfer of the framework to other settings with maintained performance levels.

Our approach also differs from the framework with the addition of a detection function. Combined detection and prediction methods are common in weather forecasting but not in infectious disease epidemiology. It is somewhat surprising that this is the case, as there are several studies that have focused on developing either influenza detection or influenza prediction algorithms, but seldom a combination of these [28]. However, one possible reason for this fact can be that researchers or research groups have attempted to develop integrated detection and prediction methods but failed to obtain satisfying results for one or several components and therefore chosen not to publish the findings. We suggest researchers in this field to publish methods even if the obtained results are not satisfying because other researchers may want to further develop and improve these methods.

### Public Health Implications

The performance evaluation of the integrated detection and prediction method based on retrospective data showed promising results. The rationale for developing our influenza detection and prediction method was to inform the planning of local response measures and adjustments of health care capacity. During emerging epidemics of infectious diseases, it is vital to have up-to-date information on epidemic trends because hospitals and intensive care units have limited excess capacity [29]. In Sweden, for example, the hospital bed capacity is habitually over-extended already before winter influenza seasons with on average 103 patients occupying 100 regular hospital bed units [30]. It is therefore important that increased influenza activity is noticed early at the local level to make time for adjusting primary care and hospital resources already under pressure to the demand in the community (especially hospitalizations requiring intensive care). Syndromic surveillance methods here serve as complements to traditional surveillance by provision of earlier indications of influenza activity [31,32]. However, although the method showed promising performance, we contend that a retrospective evaluation of a single season is insufficient for drawing valid conclusions about its effectiveness. We find that a retrospective evaluation of numerous seasons still would be insufficient. Instead, prospective evaluations are warranted where historical data are only used to determine thresholds and other parameters, and the method is applied on forthcoming epidemics.

### Strengths and Weaknesses

The method presented in this paper has both strengths and weaknesses that need to be taken into regard. An important strength is that the design rationale is documented in detail in order to allow the researchers to consider the arguments for different design decisions when building next generation of integrated detection and prediction methods. Another key strength is that analyses of an epidemic is divided into three separate components (beginning of epidemic, peak timing of epidemic, and peak intensity of epidemic), where statistical and mathematical assumptions for each of these components are made independently of each other. Also, different data sources are applied in each component. Concretely, to detect the beginning of an epidemic, exponential regression is applied on influenza-diagnosis data; to predict the peak timing, simple linear regression is applied on syndromic telenursing data; and to predict the peak intensity, the epidemic is assumed to follow a bell-shaped function of time around the peak and therefore a derivate of the normal distribution density function is applied on influenza-diagnosis data. An approach similar to this has rarely been reported in the field of influenza surveillance. One possible limitation of the method design is that the series of actual influenza-diagnosis data are smoothed and the peak of the smoothed series is used as the actual peak. However, as mentioned in the Methods section, the reason for this design choice was to reduce the risk of misleading influence from outliers.

One potential limitation concerns the use of sensitivity and specificity in the method. These metrics are, however, restricted to assess the accuracy of the alerting threshold. We have previously contended that it is important to determine the appropriate period in time which calculations of sensitivity and specificity are to be based upon [33]. This issue mainly concerns sensitivity because once an epidemic has started, it is known that the daily incidence will exceed the predetermined threshold for a certain period ahead. Expanding this period would generate higher sensitivity and thereby overestimate the method performance. Similarly, if the periods are set too short, the performance of the method may be underestimated. A short period of the specificity can also lead to a situation where hypothetic increases of the incidence level during interepidemic periods are ignored in the calculations, leading to a higher value of the specificity which can also be deceiving. Therefore, we chose to base the calculations of the sensitivity on the first 45 days of an epidemic and the specificity calculations on the period from when the previous epidemic has ended and until the beginning of the current epidemic (ie, for specificity the period length varied).

Another possible limitation concerns the second prediction component, where we chose to apply linear regression on 7-day periods for the search of positive and negative trends in order to find the peak timing in the syndromic data. The length of the period could have been extended with 1-2 days to get more reliable estimates of the trend. However, this alternative was weighted against the risk of predicting the influenza-diagnosis peak with fewer days in advance, and the advantage with earlier prediction of saving these days was preferred. Another limitation is that the prediction of the peak intensity is affected by the peak timing prediction, since a precise prediction of the peak timing increases the chance of an accurate prediction of the peak intensity. Concretely, if the timeliness for the prediction of the peak timing was 0 days instead of 7 days in our retrospective evaluation of the 2008-09 winter influenza season, the predicted peak intensity would have been estimated to 7.7 instead of 6.3 influenza-diagnosis cases/100,000 compared with the factual 8.5 influenza-diagnosis cases/100,000. In other words, the absolute difference between the predicted and the actual incidence would have been 10% instead of 26%. Finally, in the second prediction component, we assumed that an influenza epidemic takes a bell-shaped form from the beginning to the end, and therefore we employed a derivate of a normal distribution density function to find the peak intensity. The same assumption was used by Bregman and Langmuir [24] to predict the course of the AIDS epidemic in the United States but was later shown to be inaccurate [34]. However, in the case of the AIDS epidemic, the bell-shaped function was applied in a setting where the underlying premises radically differed from that at hand in the present study, that is, to predict the course of the AIDS epidemic which had been ongoing for several years in an ill-defined population. In contrast, in our study, the function is used only to find the peak intensity in an increase of influenza activity that lasts for only one season. In this context and for these purposes, we believe that the assumption of a bell-shaped curve is defensible.

### Conclusions

During the recent decade, a multitude of algorithms for influenza detection or prediction have been reported [28,35-38]. Unlike meteorology where methods for integrated very-short and long-term predictions have been used in practice settings for several decades (see eg, [39-41]), surprisingly few such approaches have been reported for influenza surveillance. Our integrated detection and prediction method is one of the first designed for application on naturally occurring local influenza epidemics. The results of this study indicate that further prospective evaluations of the method are justified.

## Abbreviations

AIDS: acquired immune deficiency syndrome
CDC: Centers for Disease Control and Prevention
EPRs: electronic patient records
ICD-10: International Classification of Diseases version 10
ROC: receiver operating characteristic
SIR: Susceptible-Infectious-Recovered
